# Current and potential uses of drones in healthcare delivery in sub-Saharan Africa: a rapid scoping review

**DOI:** 10.1080/16549716.2026.2719410

**Published:** 2026-09-01

**Authors:** Nhyira Yaw Adjei-Banuah, Abdou Moussa Ismaguel, Kezia Naa Amerley Akosua Amarteyfio, Eugene Paa Kofi Bondzie, Jean Claude Romaric Pingdwindé Ouedraogo, Irene Akua Agyepong, Aissa Diarra, Tolib Mirzoev, Angela Owusu-Ansah, Martin McKee

**Affiliations:** a Faculty of Public Health, Ghana College of Physicians and Surgeons, Accra, Ghana; b Department of Medical Anthropology, Laboratory for Studies and Research on Social Dynamics and Local Development, Niamey, Niger; c Department of Traditional Medicine and Pharmacopoeia/Pharmacy, Institute of Health Sciences Research (IRSS), Ouaga, Burkina Faso; d Department of Socio-anthropology, Laboratory for Studies and Research on Social Dynamics and Local Development, Niamey, Niger; e Department of Global Health and Development, London School of Hygiene & Tropical Medicine, London, UK; f Office of the Provost, Ashesi University, Berekuso Eastern Region, Ghana; g Department of Health Services Research and Policy, Faculty of Public Health and Policy, London School of Hygiene & Tropical Medicine, London, UK

**Keywords:** Unmanned aerial vehicles (UAVs), unmanned aerial devices, last-mile delivery, medical supply delivery, health services accessibility

## Abstract

Drones are changing healthcare delivery, particularly in sub-Saharan Africa (SSA), where traditional logistics face geographical, infrastructural, and resource limitations. This review maps where drones have been used in SSA, how they have been used, the outcomes and challenges, and the potential to expand drone systems to address other healthcare problems. A rapid scoping review methodology was employed, sourcing literature from Google Scholar, PubMed, SCOPUS, Web of Science, Cairn.info, and grey literature using targeted keywords such as ‘drones,’ ‘medicine delivery,’ and ‘sub-Saharan Africa’ to build a search query. Twenty-nine papers met the inclusion criteria, covering 12 countries, although the evidence base was heavily concentrated in Ghana and included a mix of peer-reviewed articles, technical reports, and media or organisational sources. Drone delivery systems have improved healthcare logistics across SSA by reducing delivery times, addressing rural access issues, improving maternal, neonatal, infectious disease, and emergency outcomes, and optimising supply chains. However, challenges such as payload limitations, dependency on donor funding, regulatory and policy restrictions, and workforce shortages hinder scalability. Drones have great potential for healthcare delivery in SSA, offering rapid, efficient, and cost-effective solutions that are transferable to non-communicable diseases (NCDs) and to potential and emerging applications such as vector surveillance and public health messaging. While their contributions to improving maternal health and emergency response are notable, addressing systemic issues is crucial. Drones show promise for improving healthcare logistics in underserved settings, but their long-term value depends on resolving regulatory, infrastructural, workforce, and financing constraints, and on generating stronger comparative generalisable evidence.

## Paper Context

- **Main findings:** Drone delivery systems have improved healthcare logistics across sub-Saharan Africa by enhancing emergency responses, addressing rural access issues, and optimising routine medical deliveries.
- **Added knowledge:** By highlighting the use of drones in resource-constrained settings of sub-Saharan Africa, this paper clarifies why drones are particularly relevant in this region rather than simply transferable technologies.
- **Global health impact for policy and action:** The evidence shows that drone systems can be scaled up as an intervention to strengthen health supply chains in Low- and Middle-Income Countries, provided they are integrated into national health systems and supported by broader system investments.

## Background

Drones are increasingly recognised for their transformative potential in healthcare delivery, especially in overcoming challenges faced by traditional logistics, offering rapid, efficient, cost-effective solutions in time-sensitive emergencies and logistical bottlenecks [1,2]. A 2021 review of the use of drones in healthcare delivery, drawing mainly on evidence from elsewhere, but also early studies from Ghana and Rwanda, argued that, like mobile phones, drones offer scope to overcome infrastructure weaknesses [3]. Our preliminary, non-exhaustive search of the internet, including news articles and academic publications, identified examples from across the continent.

We can identify four broad groups of drone uses in healthcare. The first group of uses is in emergency medical services (EMS) when time is critical. They are deployed to deliver time-sensitive supplies, such as automated external defibrillators (AEDs), naloxone, and blood products, which significantly improve response times. This rapid delivery capability is vital for enhancing patient outcomes in time-critical situations. Current deployments show promise, as drones can reduce the time to intervention [4]. However, further research is needed to integrate them effectively into existing EMS systems.

The second group is used to deliver care in rural and remote areas, where geographical and logistical barriers often impede traditional transportation methods. For instance, drones have proven more cost-effective and efficient in North Dakota than trucks for transporting pharmaceuticals [5]. The third group is used to deliver routine healthcare services. Drones can optimise logistics by delivering and retrieving medications and test kits for patients with chronic diseases. This may reduce operational costs while improving service efficiency when informed by models for optimising drone hub locations and routes [6]. The fourth group of uses is in pandemic response. During the COVID-19 pandemic, drones were instrumental in distributing self-testing kits, reducing human contact, and thus the risk of virus transmission [7].

Yet, challenges remain, chiefly funding, maintenance, and equitable benefit. In low- and middle-income countries (LMIC), drone systems are often seen as prohibitively expensive: governments in Ghana and Rwanda have faced criticism that funds could better strengthen existing infrastructure than create parallel, costlier systems [1,3,8,9]. Even in advanced countries, drone system costs often overshadow benefits, including environmental gains [10,11].

For decades, sub-Saharan African (SSA) countries have struggled to overcome significant challenges in ensuring access to quality healthcare, including an inequitable distribution of skilled personnel, inadequate healthcare infrastructure, and geographical barriers [8]. Drones delivering essential medicines and medical supplies are a promising solution to address these persistent access issues [1]. Indeed, as noted above, SSA has been identified as an ideal region for deploying innovative solutions like drones to tackle these systemic challenges, particularly in rural and hard-to-reach areas [3]. This review examines the feasibility of drones and their diverse applications in healthcare across SSA.

### Previous reviews of drone use in healthcare

A few reviews have examined drones’ diverse healthcare applications. Zègre-Hemsey & Cheskes [12] examined drone-supported AED delivery in the US, Sweden, and Canada, highlighting the importance of national regulatory context. Nyaaba and Ayamga [3] documented worldwide drone delivery of first aid kits, supplies, and personal protective equipment, underscoring the need for multi-sectoral commitment to sustainability. In India [13], drones have been predominantly used for emergency response and disaster response. Laksham [2] and Stierlin et al. [14] similarly highlight the potential cost and access benefits in remote areas.

In SSA, existing reviews show that drones have been used primarily in pandemic response, emergency medical supplies, vaccine delivery, and improving access to healthcare, especially emergency, maternal, and communicable disease care [1,15,16]. However, these reviews reported drone use in only a few SSA countries, and it remains uncertain whether the benefits of drone technology can be effectively extended to other areas of healthcare across the entire region. This review makes three contributions. First, it maps the current uses of drones in healthcare in sub-Saharan Africa. Second, it synthesises the reported operational, health, economic, and implementation lessons from these programmes. Third, it examines whether and how these lessons may inform broader health system applications, including for chronic disease management.

## Methods

The review methods have been registered at https://osf.io/p4jh3/overview. We conducted a rapid scoping review following Arksey and O’Malley’s [17] methodology: research question formulation, study identification, study selection, data charting, and collating/summarising results. We chose this approach because the field is rapidly emerging, our questions were exploratory, and our initial searches found sparse literature with relevant material in grey literature [18]; the evidence was heterogeneous in design, context, and timing. We therefore included heterogeneous evidence types, including organisational or media sources with unique, verifiable operational information, used to identify applications and implementation features rather than treated as equivalent to peer-reviewed outcome evaluations.

The review was undertaken to inform the design of an intervention to improve access to medicines in three West African countries [19].

### Formulating the research question

Based on our initial search of the literature, iterative consultations among the authors, and to inform intervention design, our research questions were:

- How and where have drones been used to deliver medicines and other health logistics in sub-Saharan Africa?
- What lessons can be learned from the experience of implementing drones in healthcare in sub-Saharan Africa?
- Can drones be used to address medicines and other access problems, and if so, how and where should they be used?

### Identifying relevant studies

We conducted searches in Google Scholar, PubMed, SCOPUS, Web of Science, and Cairn.info using the following keywords, their synonyms, and, where appropriate, Medical Subject Headings (MeSH): ‘drones’, ‘medical’, ‘capacity’, ‘LMICs’, and ‘sub-Saharan Africa’. The generic search strategy is shown in Table 1. The search summary tables for each database are also provided in Supplementary File 1. We searched the French-language literature using the French translations of the same search terms in Google Scholar and Cairn.info. Anglophone team members reviewed English papers, and Francophone team members reviewed French papers. References cited in the initial set of papers retrieved were searched. Grey literature was searched for iteratively using standard search engines, following leads as appropriate. Databases were searched from inception to date. The final search was conducted in February 2026.

**Table 1.** Search strategy.

Search No.	Concept	Search String
1	Drones	drone* OR ‘unmanned aerial vehicle*’ OR ‘UAV*’ OR ‘unmanned aircraft system*’ OR ‘UAS*’ OR ‘aerial delivery system*’
2	Medical	medical OR medicine OR vaccine* OR blood OR ‘laboratory sample*’
3	Capacity	efficien* OR performance* OR evaluat* OR delivery OR reliabilit*
4	LMICs / Sub-Saharan Africa	‘Sub-Saharan Africa’ OR SSA OR ‘Africa south of the Sahara’ OR LMIC* OR ‘low- and middle-income countr*’ OR ‘developing countr*’
	Final output	1 AND 2 AND 3 AND 4

Relevant organisational and industry websites, including Ministries of Health, drone technology companies such as Zipline, UNICEF, and Gavi were searched for grey literature and other sources. Contrary to the protocol specification, relevant news articles and press releases were included because they contained verifiable information for our study.

### Study selection

We focused on studies that: (1) were conducted in sub-Saharan countries where drones have been used in healthcare; (2) evaluated the outcomes of drones in improving access to medicines and health logistics; or (3) described the challenges associated with drone delivery systems. We included both single-country and multi-country studies that had SSA-specific data that could be extracted separately from other regions. Table 2 summarises our eligibility criteria using the Population Concept Context (PCC) framework. No direct author contact was made to obtain unpublished data.

**Table 2.** Eligibility criteria.

	Include	Exclude
Population	Health logistics systems, healthcare facilities, and population groups targeted for drone-based delivery.	Studies focused solely on drone engineering, mechanics, modelling, or manufacturing without a health-delivery application.
Concept	The deployment, effectiveness, and challenges of using drones to deliver medical supplies	Studies focused on non-medical drone applications (e.g. military combat, commercial shipping, agriculture, search and rescue).
Context	Geographic settings within SSA	Studies conducted in African countries north of the Sahara or non-African countries Multi-country studies including SSA but without separable SSA country-specific data
Language	English and French	Articles published in Portuguese and Arabic

**Figure 1.**
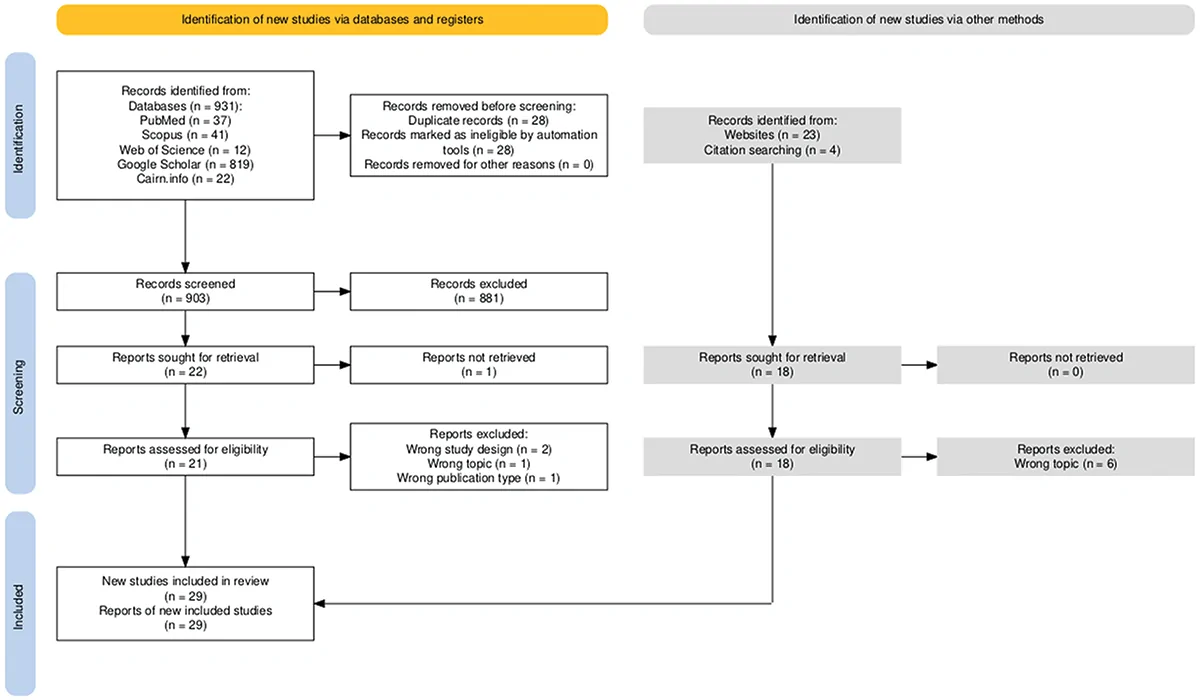
PRISMA flow diagram of the study selection process.

We exported our results to the Rayyan software to remove duplicate articles. Next, five reviewers (NYAB, AMI, KNAAA, EPKB, and JCRPO) manually screened all titles and abstracts, aided by a screening flowchart. The same five reviewers then read the full texts of the relevant papers, and those that met our eligibility criteria were included for analysis. Two authors independently screened each article, and disagreements were resolved by consensus. Language tasks were split among the team – NYAB, KNAAA, and EPKB reviewed the English articles, while AMI and JCRPO reviewed the French articles. Our final analysis included 29 papers. A PRISMA flow diagram [20] (Figure 1) shows the screening results.

The diagram illustrates the systematic workflow of the scoping review process, detailing the number of records identified from each database; records screened, sought for retrieval, and assessed for eligibility; and records included in the analysis. The workflow for records identified, screened, and included from websites and through citation searching is also illustrated.

### Charting the data

Data were extracted by MM, IA, NYAB, AM, KNAAA, EPKB, and JCRPO into a data extraction form. Data from each paper were charted independently in Microsoft Excel by two reviewers; discrepancies were resolved through discussion. Only information relevant to our research objectives was extracted. This included citation information, countries studied, study characteristics, context and setting, drone intervention details, reported outcomes, and implementation experiences. Details of these broad categories and the extracted data are provided in Supplementary File 3. Critical appraisal was not undertaken, consistent with scoping review methodology.

### Collating, summarising, and reporting the results

The results were summarised narratively in line with the research questions, supported by tables and figures. Themes were determined a priori based on expert consensus and literature: (a) where drones have been used, (b) how drones have been used, (c) implementation challenges of drone use in healthcare in SSA, and (d) descriptive reports of outcomes and impacts – sub-themes, identified through analysis, aided in summarising and reporting the results. The main categories of drone use were compared by country. Thematic analysis was conducted in NVivo version 14, and other analyses in Microsoft Excel.

## Results

### Search results and study profile

The searches identified 931 records. After removal of 28 duplicates, 903 titles and abstracts were screened. Twenty-one full texts identified through database searches were assessed; 17 met the inclusion criteria. Citation tracking, website searches, and grey literature searches yielded a further 27 records; 18 were reviewed in full text, and 12 were included. The final review, therefore, comprised 29 papers, as shown in the PRISMA flow diagram (Figure 1) [20]. Of these, 22 were peer-reviewed journal articles, four were technical reports, and three were press releases or news reports from credible organisations.

The evidence base was geographically uneven. Ghana accounted for 16 studies [8,21–35], followed by Madagascar [36–38], Senegal [27,36,38] and Malawi [8,36,39] (3 each), Rwanda [40,41], Nigeria [27,42], Côte d’Ivoire [27,43], and Cameroon [26,44] (2 each), with one study each from the Democratic Republic of the Congo (DRC) [45], Guinea [46], Uganda [47], and Kenya [48]. Because several sources reported on more than one country [8,26,36], these figures are country-level observations, not unique studies; the 29 included studies generate 37 country-level data points in total. Methodologically, the included studies were heterogeneous: 12 were quantitative [22–24,27,30–32,34,37,38,40,41,46], 15 qualitative [8,21,25–28,33,36,39,42–44,47,48], and 2 were mixed methods [29,45]. Most were conducted in remote rural settings, reflecting the logistical niche in which drones have so far been deployed. The predominance of Ghana reflects the scale and longevity of the Zipline public–private partnership, which has generated a wider range of evaluations than programmes elsewhere (see Box 1).

Box 1. A case study from Ghana.

Ghana, a lower-middle-income country in West Africa, faces persistent challenges in healthcare delivery, especially in rural areas, where nearly 60% of the population lives. These areas often lack adequate health infrastructure and personnel, contributing to healthcare disparities [1,30,34].
To address this, the government partnered with Zipline in 2019 to deliver medical supplies via drones [33]. By 2021, six distribution hubs served over 2,200 facilities and 12 million people [30]. Zipline’s fixed-wing drones, with a 1.75–2 kg payload and 210 km range, deliver supplies within 30 minutes using parachute drops [1,34]. However, their lack of VTOL capability limits item retrieval ability [1].
Operations focus on emergency deliveries, routine supply distribution, and stock replenishment [25]. Drones have delivered antimalarials, vaccines, and blood products, and have been used to transport COVID-19 test samples [29]. Their use has been linked to increased antenatal visits, reduced maternal mortality, and improved vaccine availability [29].
Impact assessments show drones enabled nearly 15,000 additional immunisations in 2021, prevented 688 disease episodes, and saved four children’s lives [32,34]. In Ghana’s Western North region, drone vaccine delivery was estimated to have saved 727 lives and reduced healthcare costs [32].
However, challenges remain. First, reports of medication stockouts at distribution centres suggest that incorporating NCD supplies into drone delivery systems could exacerbate pre-existing supply chain issues and delay care [25]. Adding NCD supplies to the existing system could strain the infrastructure unless the system is expanded to accommodate increased demand. Second, primary care workers can find coordinating medication orders and deliveries burdensome [8]. Training dedicated non-clinical personnel to manage these logistics could alleviate this issue. Third, Zipline’s recruitment of clinical staff has contributed to internal brain drain [8].

This uneven evidence base matters. Drone evidence comes from a small number of early-adopting countries and operational models, especially public–private partnerships with major international providers. The review, therefore, maps an emerging field, and its findings are descriptive and hypothesis-generating, rather than definitive estimates of effect. A summary of included studies is in Table 3.

**Table 3.** Summary of included studies.

Full citation, country	Evidence type & Study design	Drone type	Payload capacity & Flight range	Purpose/application / Operational model	Summary of Findings
Kremer P, Haruna F, Briegleb C, Amoah ME, Oteng KF, Boadi S, et al. A mixed-method impact assessment of the use of aerial logistics to improve maternal health and emergencies outcomes in the Ashanti Region of Ghana. BMC Health Services Research 2025 25:1. 2025–03-17;25(1). https://doi.org/10.1186/s12913-025–12479-1. Ghana	Empirical: Mixed-methods	Autonomous medical drones	Not mentioned	Emergency blood products, oxytocin, medications for eclampsia, snake antivenom, rabies shots, injectable medicine for severe malaria. Government contracted private technology provider (Zipline).	Deliveries typically within 5–30 minutes, curbing referrals and saving lives via timely emergency medication. Antenatal visits increased 20% (RR 1.20), and referrals increased 3.41-fold; outpatient visits rose 20.8%, with CHPS compounds seeing a 78.1% increase. Facility deliveries increased 26% and maternal deaths fell 56% (RR 0.44); emergency visits fell 4%. No significant change in hysterectomies or neonatal deaths. Reduced transportation and stocking costs; reduced wastage and stockouts. 100% patient satisfaction; 90% rated facility services as ‘much better.’ Barriers included facility-level policy restrictions, medication shortages, and poor roads/electricity.
Kremer P, Haruna F, Tuffour Sarpong R, Agamah D, Billy J, Osei-Kwakye K, et al. An impact assessment of the use of aerial logistics to improve access to vaccines in the Western-North Region of Ghana. Vaccine. 2023;41(36):5245–52. Epub 20,230,619. https://doi.org/0.1016/j.vaccine.2023.06.036. PubMed PMID: 37,344,263. Ghana.	Empirical: Quantitative (Retrospective quasi-experimental and cross-sectional survey).	Autonomous medical drones	Not mentioned	Vaccines (National Immunization Programme), blood, and medical products. Government contracted private technology provider (Zipline).	Vaccine access achieved in under 60 minutes. Vaccination coverage increased 13.1–37.5% points; 21% expansion in patient vaccinations; 91.1% of providers perceived improved access. Infectious diarrhoea (ages 5–9) fell 41.6%, ~430 fewer cases/year, ~727 potential lives saved. Stockout duration fell 30%; missed vaccination opportunities fell 44%. Provider satisfaction averaged 8.6/10 (served) vs 7.8/10 (non-served). Barriers: weak cold chain systems, limited infrastructure, inaccurate denominator estimates.
Damoah IS, Ayakwah A, Tingbani I. Artificial intelligence (AI)-enhanced medical drones in the healthcare supply chain (HSC) for sustainability development: A case study. Journal of Cleaner Production. 2021;328:129598. Ghana.	Empirical: Qualitative case study	AI-enhanced autonomous medical drones	50 miles radius	Vaccines, blood, and life-saving medical supplies for childbirth, accidents, and snakebites. Government-contracted private technology provider (Zipline).	Deliveries under 30 minutes versus 4.5 hours by road; reduced reliance on slower vehicle-based delivery. Universal access to lifesaving medicine for remote communities; reduced overcrowding at larger hospitals. Reduced infant/maternal mortality. Cost reductions via elimination of overstocking and lower overhead; reduced wastage; 24-hour supply chain. Health worker job satisfaction and motivation; patient gratitude for timely delivery. Noise-free, carbon-free operation with minimal vegetation disruption. Improved local employment and skills via training (USA, Rwanda) and recruitment of Ghanaian engineers/pharmacists. Enabled by presidential support and centralised storage; required per-flight aviation clearance.
Knoblauch AM, de la Rosa S, Sherman J, Blauvelt C, Matemba C, Maxim L, et al. Bi-directional drones to strengthen healthcare provision: experiences and lessons from Madagascar, Malawi and Senegal. BMJ Glob Health. 2019;4(4):e001541. Epub 20,190,730. https://doi.org/10.1136/bmjgh-2019-001541. PubMed PMID: 31,413,873; PubMed Central PMCID: PMC6673761. Madagascar; Malawi; Senegal.	Empirical: Qualitative (SWOT analysis and descriptive experience sharing of pilot projects)	Hybrid (fixed wing and quadcopter); Quadcopter.	Payload: Up to 1.5 kg (Madagascar) 1 to 6 kg (Malawi) 2 kg (Senegal) Range: 60 km (Madagascar) 80 km to 100 km (Malawi) 60 km (Senegal).	Sputum/medication transport for TB (Madagascar); TB/HIV diagnostic samples, viral load, medication, blood, injectable oxytocin (Malawi); essential drugs and medical samples (Senegal). NGO-led pilots in collaboration with Ministries of Health.	Main perceived benefit was potential transport time saved, with shorter delays in sample referrals. Increased equity and access for hard-to-reach facilities. Cost per km was higher for drones than motorcycles (Malawi), though modelling suggested savings if usage were maximised. Reduced stockouts; optimised specimen referral system. High acceptability in Madagascar and Malawi post-introduction, though with privacy/safety concerns about cameras. Challenges included high-energy lithium battery shipment rules, delayed regulatory development, and limited real-world technology readiness (e.g. GPS interference). Rwanda’s performance-based regulatory model and the African Drone and Data Academy were cited as key enablers/sustainability factors.
Ospina-Fadul MJ, Kremer P, Haruna F, Adomako-Boateng F, Oteng KF, Tsali DN. Cost-Effectiveness of Aerial Logistics for Maternal and Newborn Health: A Simulation-Based Analysis Grounded in Real-World Evidence from the Ashanti Region in Ghana. Journal of Health Economics and Outcomes Research. 2025 Sep 17;12(2). doi: https://doi.org/10.36469/001c.143065. Ghana.	Modelled: Microsimulation modeling and cost-effectiveness analysis, anchored in empirical data	Autonomous medical drones	Not mentioned	Routine maternal health commodities (nutritional supplements, vaccines, oxytocin, blood/plasma). Aerial logistics for routine service delivery anchoring supply chain responsiveness.	Improved supply chain responsiveness reduced missed treatment opportunities. Modelled 2,252 additional women reaching ≥8 ANC visits and 363 additional women reaching ≥1 visit. Averted 3,754.99 DALYs and 130 premature deaths; reduced neonatal mortality and sepsis. ICER of US$106.79/DALY (government), US$2.24/incremental ANC visit, US$5.60/additional facility birth. Solved chronic stockouts of maternal commodities; consistent availability increased trust in the health system and ANC schedule completion.
Dzisi EK, Patterson MK, Iddrisu S. Drones in healthcare logistics: Insights from healthcare professionals’ perspective on Zipline delivery services in Ghana. Dialogues in Health. 2026;8(100285.). doi: https://doi.org/10.1016/j.dialog.2026.100285. Ghana.	Empirical: Quantitative (Cross-sectional survey).	Autonomous medical drones	100 km radius	Blood products, vaccines, routine medical supplies, emergency items, and laboratory samples. Government-contracted private technology provider (Zipline).	66.9% of staff reported improved emergency response times; 80.9% affirmed timely delivery. Service reached ~12% of designated hard-to-reach communities. 56.4% reduction in maternal deaths in Ashanti Region drone-supported facilities. Government debt of ~$16 million raised value-for-money concerns. 54.1% perceived improved supply delivery; 33.1% agreed drones reduce supply chain gaps. 62.2% awareness and 36.3% adoption among workers; 41% identified security concerns. Barriers: high operational costs (68%), technical limitations e.g. battery/payload (55.3%), regulatory hurdles (35.6%), and gaps in facility-level training/sensitisation.
Nisingizwe MP, Ndishimye P, Swaibu K, Nshimiyimana L, Karame P, Dushimiyimana V, et al. Effect of unmanned aerial vehicle (drone) delivery on blood product delivery time and wastage in Rwanda: a retrospective, cross-sectional study and time series analysis. Lancet Glob Health. 2022;10(4):e564-e9. doi: https://doi.org/10.1016/s2214-109x(22)00048–1. PubMed PMID: 35,303,465. Rwanda.	Empirical: Quantitative (Retrospective, cross-sectional study and interrupted time series analysis).	Servo-electric launched autonomous medical drones	80 km radius (22,500 km^2^ coverage).	Blood and blood products (red blood cells, negative rhesus blood, fresh plasma, platelets). Government collaboration with private company (Zipline) for autonomous aerial delivery.	Mean delivery time of 49.6 minutes, 79–211 minutes faster than estimated road delivery. Both hubs covered 90% of the country outside the capital; 12,733 orders delivered over 32 months. Blood unit expirations fell by 7.1/month, a 67% reduction at 12 months. Drone distribution was costlier per unit than traditional methods but reduced stockouts of rare groups and platelets, reduced expired products, and digitised the supply chain. Initial public reaction was a mix of admiration and scepticism. Fewer than 1% of units were damaged in transit. Enabled by a contractual performance framework (<1 hr emergency, <4 hr resupply); barriers included remote/poor-road locations and unpredictable demand fluctuation; required blood bank infrastructure investment at facility level.
Ospina-Fadul MJ, Kremer P, Stevens SE, Haruna F, Okoh-Owusu M, Sarpong GK, et al. Cost-Effectiveness of Aerial Logistics for Immunization: A Model-Based Evaluation of Centralized Storage and Drone Delivery of Vaccines in Ghana Using Empirical Data. Value in Health Regional Issues. 2025/03/01;46. doi: https://doi.org/10.1016/j.vhri.2024.101066. Ghana.	Modelled: Attack rate modelling and incremental cost-effectiveness evaluation (Monte Carlo simulations), anchored in empirical data.	Autonomous medical drones	Not mentioned	Full immunization vaccines (Pentavalent, PCV, MR, BCG); centralized storage and on-demand delivery. At-scale aerial logistics integrated with the Expanded Program on Immunization (public-led).	Timely delivery ensured consistent vaccine availability and reduced family travel burden. An additional 14,979 full immunisation courses were completed, with significant district-level coverage improvement. Averted 688 disease episodes and >170 discounted DALYs; saved 4 children’s lives in study districts in 2021. ICER of $58/DALY (government) and $41/DALY (societal); $0.66 per incremental fully immunised child; $0.27 last-mile delivery cost per dose. Reduced wastage and stockouts; cost-saving beyond ~20,807 replacement doses. Reliability strengthened caregiver trust and satisfaction with vaccine access. Context: ambiguous quality of traditional ground logistics data and COVID-19 disruptions; modelling anchored to NHIS treatment costs and free EPI disease treatment policy.
Wang N. ‘As It Is Africa, It Is Ok’? Ethical Considerations of Development Use of Drones for Delivery in Malawi. IEEE Transactions on Technology and Society. 2021;2(1):20–30. Malawi.	Empirical: Qualitative (Case study)	Semi-automatic, can vertically take off and land (VTOL)	Up to 6 kg	Laboratory samples (HIV/AIDS, TB), blood products, vaccines, and medical supplies. International aid agency and contracted drone company in collaboration with the Government of Malawi.	Delivery time fell from 6–8 hours (boat) to 1 hour each way; sample turnaround fell from 5–8 weeks to under 4 weeks, reducing pressure on lab capacity. Connected underserved islands to mainland health logistics. Patient care appeared to improve but remained donor-dependent; cost-effectiveness in this development context was largely unknown given high capital/maintenance costs. Stable sample inflow caused staff overburdening and machine downtime; supply was interrupted during crashes. Mixed acceptance, including sceptical views favouring facility capacity investment over drones. One crash into Lake Malawi (wind-related); safety, environmental, and patient confidentiality risks were raised. Enabled by the Humanitarian Drone Testing Corridor (2017) and relaxed airspace rules; barriers included headwinds, poor connectivity, lack of insurance, donor dependence, and limited local spare parts/training capacity.
Bahrainwala L, Knoblauch AM, Andriamiadanarivo A, Diab MM, McKinney J, Small PM, et al. Drones and digital adherence monitoring for community-based tuberculosis control in remote Madagascar: A cost-effectiveness analysis. PLoS One. 2020;15(7):e0235572. Madagascar.	Modelled: Cost-effectiveness analysis	Hybrid (quad-copter and fixed-wing)	Payload: 2.2 kg Range: 120 km	Sputum samples and TB medication. Research/NGO (Stop TB Partnership project).	Faster diagnosis, increased patient retention, and reduced transportation challenges for a population of ~200,000. TB-related deaths fell from 51.3% to 19.0% in the model; 2,514 DALYs averted; 61.2% increase in case finding and treatment initiation versus standard DOTS. ICER of $177/DALY averted. High start-up costs for drone systems and digital adherence technology (evriMED), and difficulty in bad weather/limited infrastructure were noted barriers. Required specialised drone personnel and CHW training; 7 round-trip flights needed per full 6-month TB therapy course.
Ghana’s Medical Drone Delivery System takes off [Internet]. Accra: Ministry of Health, Ghana; 2019 [cited 2026 Jul 20]. Available from: https://moh.gov.gh/ghanas-medical-drone-delivery-system-takes-off/. Ghana	Grey literature: Press release (non-evaluative)	Not mentioned	Not mentioned	Medical supplies, blood, critical medicines, vaccines, snake antivenoms. Public–Private Partnership (PPP).	Designed for shortest-possible-time, 24-hour delivery, potentially reaching over 15 million people and 2,000 health facilities. Framed as preventing maternal deaths and saving lives from snakebites/accidents, with significant decreases in supply chain wastage. Enabled by government commitment to social inclusion and use of technology for national development, led by the Office of the Vice-President.
Bu D, Hernandez M, Haruna F, Abasi PM, Kremer P. Improving Health Access Through the Distribution of COVID-19 Vaccines Using Drones in Ghana 2024 [cited 2024 4th December]. Available from: https://ssrn.com/abstract=4401693 or http://dx.doi.org/10.2139/ssrn.4401693. Ghana.	Empirical: Quantitative (Retrospective, ecological study)	Autonomous medical drones	Payload: 2 kg Range: 210 km (round-trip)	COVID-19 vaccines. Government contracted private technology provider (Zipline).	2.5 million doses delivered, with proportionally more reaching socioeconomically disadvantaged and remote areas (pro-equity distribution). UAV transport/logistics savings offset implementation costs even at low payloads (0.4 L); reduced functional difficulty of reaching remote areas and enabled contactless, infection-chain-breaking delivery. Required significant engagement with regulators and communities; raised labour market concerns regarding displacement of traditional transport logistics jobs. Relationship between disadvantage and drone delivery was stronger in rural than urban areas.
Ninsiima M, Migisha R, Ndyabakira A, Katana E, Aanyu D, Kabami Z, et al. Strategies Utilized During Sudan Virus Disease Outbreak Response in Kampala City, Uganda, 2022 − 2023. Journal of Epidemiology and Global Health. 2025;15(1):92. Uganda.	Empirical: Qualitative (After Action Review)	Not mentioned	Not mentioned	Disseminating public health messages (risk communication and disease prevention). Public (led by KCCA) with implementing partner support.	Helped reach diverse urban populations and contributed to containment of the 2022 SVD outbreak (18 confirmed cases, 2 deaths managed). Limited financial support from the KCCA treasury meant the response was largely funded by implementing partners (WHO, UNICEF, CDC, IDI) via PEPFAR/US CDC funding.
Sylverken AA et al. (2022). Using drones to transport suspected COVID-19 samples; experiences from the second largest testing centre in Ghana. PLoS One, 17(11): e0277057. Ghana.	Empirical: Quantitative (Case study)	Fixed-winged	100 km	Suspected COVID-19 samples (swabs). Public-Private Partnership (Zipline and Government of Ghana).	Average drone transport time of 39 minutes versus 117 minutes by road (*p* < 0.001) across 2,537 samples and 440 deliveries from 10 districts. Facilitated early diagnosis and rapid isolation of 560 positive cases; saved human/material resources, helping smaller districts conserve resources and enabling single-sample dispatch without batching delays. Health workers expressed satisfaction with reduced transport time, and contactless delivery increased staff social distancing. WhatsApp was used for real-time order tracking. Adverse weather (rainy season) was the main barrier, requiring land transport backup; dedicated trained personnel supported repackaging and lab handling.
Tetteh A, Addai F, Asihene WD. Investigating drone technology in health-care products delivery in rural communities in Ghana: benefits, barriers and perceptions. Journal of Humanitarian Logistics and Supply Chain Management. 2025. Ghana.	Empirical: Quantitative (Survey)	Not mentioned	Not mentioned	Vaccines, blood, anti-snake venom, and other emergency medicines. Private-sector/Government Partnership.	Reduced delivery times from hours to minutes were rated as a high benefit (mean 4.51), serving 2,300 facilities via 6 centres and improving emergency response (mean 4.43), with 1.7 million lifesaving products delivered. Project cost totalled $12 m, with reduced inventory/transportation costs cited as benefits, alongside optimised inventory management and reduced waste/stockout risk. Workers held a positive attitude toward drones (mean 4.42) and perceived reduced carbon footprint (mean 4.11). Key barriers: difficulty flying in bad weather (mean 3.22), limited infrastructure (mean 3.21), restricted payload capacity (mean 3.18), and lack of trained personnel (mean 3.13). GCAA regulations exist but require updating; workers recommended being included in decision-making.
Burchardt M, Umlauf R. Where is the bottleneck? Drones and the paradoxes of digitising medical supplies in Ghana’s landscapes of care. Global Public Health. 2023;18(1):2274434. Ghana.	Empirical: Qualitative (Ethnography)	Fixed-wing	15–30-minute delivery window	Emergency medicines, blood, vaccines. Private start-up contract with state.	The system aimed for 100% uptime and was observed to outperform land transport for far-flung clinics and CHPS compounds. However, impact was clouded by routine misuse of emergency-order allocations for non-urgent items early in the month, leaving facilities unable to use the system for true emergencies later on. Health professionals questioned whether the small number of genuine emergencies justified the financial investment; drone use was only cost-effective outside contractual emergency limits. Created parallel supply chains that revealed structural shortages of medical products upstream. Health workers were impressed but frustrated by added ordering workload. Built-in redundancy (dual propellers, backup parachutes, generators/UPS) maintained service even during infrastructure failures. Barriers included frequent stock-outs at distribution centres, electricity/network disruptions, the added time burden of ordering via WhatsApp/phone, and no clear definition of ‘emergency’ (80% of monthly deliveries were non-emergency items).
Jeon HH, Lucarelli C, Mazarati JB, Ngabo D, Song H. Last-mile delivery in health care: drone delivery for blood products in Rwanda. Available at SSRN 4,214,918. 2022. Rwanda.	Empirical: Quantitative (quasi-experimental, difference-in-differences)	Fixed-wing	Payload: 2 kg Range: 80 km radius	Blood products (RBCs, Platelets, FFP, Cryoprecipitate). Public-Private Partnership.	Lead time fell from several hours to 5–50 minutes, though benefits were concentrated among the 29 hospitals closest to the two drone ports (Muhanga and Kayonza), which were best able to expand their product assortment. PPH mortality fell 51% and trauma mortality fell 30%, with null findings for placebo conditions. On-hand RBC inventory fell 63% and RBC wastage fell 40%, reflecting a substitution effect toward as-needed platelets/FFP better aligned with clinical guidelines. Enabled by battery technology improvements; barriers included restrictive storage requirements for non-RBC products and a 12-month regulatory negotiation process with the Rwanda Civil Aviation Authority. Health improvements were concentrated among hospitals closer to the drone port.
Burchardt M, Ameso E. Bloodstream: notes towards an anthropology of digital logistics in healthcare. Anthropology & Medicine. 2024–7-2;31(3). doi: https://doi.org/10.1080/13648470.2024.2378731. Ghana.	Empirical: Qualitative (Ethnography)	Fixed-wing	Not mentioned	Blood, emergency medicines, and vaccines. Public–Private Partnership.	Drones accelerated deliveries while bypassing terrestrial hurdles, serving over 3,200 health facilities nationally, with the potential to reduce maternal mortality. However, the system sometimes failed to improve care due to lack of stock at distribution centres (‘connectivity without stock’). The Ministry of Health paid US$12.5 million in the first contract term, with distribution centre infrastructure funded by national/international donors; families paid 300–500 Ghanaian cedis per pint of blood, rising to 200–600 cedis amid commercialised blood donation driven by shortages. Health workers were initially enthusiastic but became frustrated by stock-outs, and communities expressed fears over blood quality/screening. Barriers included chronic blood shortages, distribution-centre stock-outs, and lack of electricity at CHPS compounds; the National Blood Service Act restricts drone use to emergencies only.
Ameso EA. Digital entanglements: Medical drones in African healthcare systems. Glob Public Health. 2024;19(1):2405987. Epub 20,241,002. doi: https://doi.org/10.1080/17441692.2024.2405987. PubMed PMID: 39,359,019. Ghana; Malawi.	Empirical: Qualitative (Multi-site ethnography)	Not mentioned	Not mentioned	Malawi: transportation of vaccines, laboratory samples, and delivery of laboratory reagents/results. Ghana: government contracted private technology provider (Zipline); Malawi: experimental.	Deliveries occurred within minutes, but the study highlighted workforce frustrations: drone services exposed existing rural staff shortages, as health workers struggled to juggle routine duties with order coordination, and an internal brain drain emerged as drone companies recruited experienced health professionals at higher salaries (legally required to staff distribution centres), worsening facility-level shortages. Health workers also bore added task burdens, including walking to landing sites and waiting up to 30 minutes for drone arrival. WhatsApp supported order coordination. Malawi’s African Drone and Data Academy (ADDA), supported by UNICEF, was established to train a new licensed drone-pilot workforce as part of the digital transformation of healthcare delivery.
Ecole de Sante Publique. Évaluation Des Performances Des Drones Pour Le Transport Des Vaccins Et Autres Produits De Santé Vers Les Établissements De Santé (es) Éloignés Province De L’equateur, République Démocratique Du Congo [Evaluation of Drone Performance for Transporting Vaccines and Other Health Products to Remote Health Facilities, Équateur Province, Democratic Republic of the Congo]. Kinshasa : Universite de Kinshasa 2022. Democratic Republic of the Congo.	Empirical: Mixed-methods (Quasi-experimental single group study)	Hybrid with VTOL capacity (Bidirectional)	Covers 37,345 square km network	Vaccination supplies (vaccines, syringes, diluents, adapters), returning with laboratory samples (polio, yellow fever, measles, monkeypox, Ebola, HIV, TB, COVID-19) and reports (SNIS, MAPEPI, IPV Form 1), medicines, PPEs. Public–Private Partnership (MoH and Swoop Aero).	PFA sample arrival within 2 days rose from 35% to 69%; vaccine collection time fell from 2+ days (65% of sites) to under 2 hours (100% of sites). Vaccine availability rose from 65% to 98%; DPT3 coverage reached 90.8% and VAR1 89.4%. Reduced pediatric disease potential and eliminated transport-related vaccine losses. Direct transport costs for vaccine collection fell to zero. Penta, VAR, and VAA shortages were eliminated or sharply reduced; stock availability in line with plan rose from 32% to 98%. 98.2% of community respondents were satisfied; 84.2% reported increased confidence in the health system. Eliminated river-navigation risks for health workers and freed up time for clinical activities. Enabled by government leadership and VillageReach-MoH partnership; barriers included late flight authorisations, COVID-19 border restrictions, and a 5-month health worker strike; required monthly flight authorisations and ongoing site-level training.
Eyenga GM. Dynamiques Et Mutations D’Utilisation des Drones en Afrique [Dynamics and Shifts in Drone Use in Africa]. Drones et sécurité en afrique: opportunité ou menace? [Drones and Security in Africa: Opportunity or Threat?] 012&013 ed. Cameroon: l’Ecole Internationale des Forces de Sécurité (EIFORCES); 2022. p. 45–56. Cameroon; Ghana.	Grey literature: (Ethnographic case study)	Solar-powered drones (Tagus); Autonomous fixed-wing drones (Zipline).	5 kg (Tagus ‘Akeva Care’)	Medical equipment, vaccines (COVAX), blood, medicines, COVID-19 samples, and facial recognition. Private (Tagus); Public–Private Partnership (Zipline).	COVID tests were delivered in under 1 hour, with delivery times generally reduced from hours to minutes, serving ~2,000 hospitals/health centres in Ghana. Outcomes included saved lives (maternal health, snakebites) and reduced vaccine wastage and stockouts, alongside increased diversity of available products. Zipline’s monthly fee (~$80,000) was paid via CSR funding. Perception among medical staff was generally positive, though civil society raised concerns about ‘leapfrogging’ and data sovereignty/surveillance. Enablers included government support and solar innovation (Tagus) and successful crowdfunding; barriers included administrative delays (Cameroon) and lack of specific local legislation/support for local companies.
Njoya S. Zipline authorized to deploy drone delivery services in Côte d’Ivoire 2023 [cited 2025 13th July]. Available from: https://www.wearetech.africa/en/fils-uk/news/public-management/zipline-authorized-to-deploy-drone-delivery-services-in-cote-divoire. Côte d’Ivoire.	Grey literature: Organisational/media report (non-evaluative)	Autonomous medical drones	Not mentioned	Delivery of vaccines, drugs, and blood products. Public–Private Partnership.	Announced instant logistics with planned coverage of 1,000 health facilities countrywide, beginning with a first hub in Daloa. Framed as job creation for the local population, requiring a Certificate of Automated Aircraft Operations (CEAT) from the National Civil Aviation Authority (ANAC), with local talent to be hired for distribution centre management. Presented as an expansion of prior Zipline successes in Rwanda, Ghana, Nigeria, and Kenya.
Chenal J, Ciriminna C, Jaligot R, Ginisty K, Rudaz F, directors. L’utilisation du numérique dans le contexte des villes de l’Afrique de l’Ouest [Digital Technology Use in the Context of West African Cities][Internet]. Lausanne: Centre Excellence in Africa, EPFL; 2021 Jul [cited 2026 Jul 20]. Available from: https://www.aimf.asso.fr/wp-content/uploads/2022/07/Etude-EPFL-Villes-et-numerique-Afrique-de-lOuest.pdf. Côte d’Ivoire; Senegal; Ghana; Nigeria.	Grey literature: Policy analysis	Not mentioned	Not mentioned	Blood, medicine, and vaccine delivery; urban environmental monitoring. Public (Rwanda); Private (Startups).	Drone/digital delivery was linked to a 30% reduction in infant mortality via SMS services and the broader potential for life-saving rapid delivery. High data costs were noted, with drones seen as a way to reduce infrastructure investment costs; data privacy concerns were significant (78% of users concerned). Drones were also used for environmental problem identification (e.g. water pollution/leaks). Enablers included political will, expanding 3 G/4 G coverage, and innovation hubs (AfriLabs); barriers included weak infrastructure, high internet costs, lack of legal data-protection frameworks, and a risk of digital divide/social segregation. Country readiness varied, from strong digital strategies (Group 1) to weak interest (Group 3).
Atangana DE. L’Encadrement de l’Usage des Drones en Droit Camerounais [The Legal Framework for Drone Use in Cameroonian Law]. In: Drones et sécurité en Afrique: Opportunités ou menaces? [Drones and Security in Africa: Opportunities or Threats?]. EIFORCES; 2022. p. 123–138. Cameroon	Grey literature: Policy analysis	Fixed-wing and commercial types.	Not mentioned	Security surveillance, mapping, agriculture, and potential medical use. Public (military) and Private (individuals).	Focused on legal/governance framing rather than health outcomes; noted that training costs represent 12–14% of fighter-aircraft price and that flight-hour costs are lower than piloted planes. Highlighted privacy concerns (paparazzi effect), fears of terrorist misuse, lithium battery fire risks, potential mid-air collisions, and environmental pollution. Enablers included African Union recommendations and technology transfer policies; barriers included regulatory voids, high costs, limited battery autonomy (~40 minutes for commercial units), human error, and communication problems, alongside rivalry between civil aviation authorities and private drone users.
Marti R, Teillet CR, Hobiniaina Anthonio, Fournet F. Liens entre moustiques vecteurs et environnement : apport des méthodes de télédétection satellite [Remote Sensing and Spatial Modelling: Applications to the Surveillance and Control of Mosquito-Borne Diseases]. 2022. Madagascar; Senegal.	Modelled: Remote-sensing environmental analysis	Not mentioned	Not mentioned	Mapping mosquito breeding sites and characterizing environment. Research.	Drones can improve understanding of disease transmission risk by using drone-mounted sensors to support satellite-based environmental mapping. This approach can result in more precise mapping outcomes. High cost of airborne/spatial data acquisition was noted as a barrier in some countries, alongside cloud cover affecting optical sensors and the need for expert data-processing skills (geographers, geomaticians, modellers).
Inghels M, Mee P, Diallo OH, Cissé M, Nelson D, Tanser F, et al. Improving early infant diagnosis for HIV-exposed infants using unmanned aerial vehicles for blood sample transportation in Conakry, Guinea: a comparative cost-effectiveness analysis. BMJ Glob Health 2023;8(e012522). doi: https://doi.org/10.1136/bmjgh-2023-012522. Guinea.	Empirical: Quantitative (Comparative cost-effectiveness analysis)	Not mentioned	Not mentioned	Delivery of medical products. Not mentioned.	Average round-trip time was 106 minutes (UAV) versus 213 minutes (motorcycle), saving 35.8 minutes on average and up to 109 minutes for the most remote centres (>50 km); no time was gained within 3 km of the laboratory. A higher proportion of results were delivered same-day with UAVs (47.4% vs 42.4% motorcycle, vs none at baseline). Over 5 years, UAV and motorcycle strategies saved a cumulative 834.8 and 794.7 life-years respectively versus baseline. UAV strategy cost an additional US$11.50 per infant tested versus motorcycle. Cost-effectiveness was highly dependent on UAV speed advantage, weather inoperability, UAV loss risk, number of children infected, and UAV purchase price.
Samuel L. Flying malaria vaccines reach isolated Nigerian communities: VaccinesWork; 2025 [cited 2026 10 June 2026]. Available from: https://www.gavi.org/vaccineswork/flying-malaria-vaccines-reach-isolated-nigerian-communities/. Nigeria.	Grey literature: Organisational/media report	Autonomous cargo drones	Covers health facilities scattered across a 38,000 square-kilometer radius.	Routine and on-demand delivery of childhood malaria vaccines, along with primary health commodities. Zipline International (working alongside local authorities).	Shipping times fell to 15–45 minutes regardless of weather or river terrain, expanding routine immunisation access to community health workers in marginalised riverine zones. Vaccine stock-out rates fell below 1%, protecting vulnerable infants from clinical malaria. Cold-chain integrity was maintained without long boat journeys for supply collection. Programme was celebrated locally as a ‘game-changer’ and successfully identified ‘zero-dose’ child clusters via data-driven mapping prior to launch. Enabled by close collaboration with the Bayelsa State Government, Gavi funding, and integration into official immunisation schedules; required embedding community health workers in remote settlements to receive aerial drops, overcoming geographic isolation from rivers/swampy terrain.
Maina J. How drones are transforming Kenya’s healthcare landscape: Fair Planet; 2024 [cited 2025 13th July]. Available from: https://www.fairplanet.org/story/drones-provide-health-supplies-in-africa-kenya/. Kenya.	Grey literature: Field/media report	Autonomous cargo and delivery drones	12 km − 60 km routes	Emergency delivery of blood, anti-venom, vaccines, and HIV medication. Partnerships involving international foundations (e.g. Elton John AIDS Foundation) and private firms.	Drastically reduced travel time, cutting 60 km trips from 1 hour to 10 minutes, and connecting central warehouses to remote facilities 0–95 km away. Reduced life-threatening delays in treating snakebites and obstetric emergencies, and reduced facility stockouts via on-demand delivery. Favoured by medical staff, reducing the need to close clinics for supply retrieval. Secondary uses included agricultural monitoring and site security. Enabled by proactive county government partnerships and zero-rated import taxes on drones; barriers included limited local skilled personnel and complex regulatory approvals requiring KCAA certification.
Eyenga, G.M., 2023. Une gouvernance sanitaire agile? L’expérience des drones médicaux dans la gestion de la pandémie du Covid-19 au Ghana [Agile Health Governance? The Experience of Medical Drones in Managing the COVID-19 Pandemic in Ghana]. *Cahiers d’études africaines*, (250), pp.315–341 (pp. 1–2). Ghana	Empirical: Qualitative (Ethnography)	Fixed-wing (Zipline)	Payload: 1.7 kg Range: 70 km	Delivery of COVID-19 and other test samples, COVID-19 vaccines	Delivery time cut to under an hour. Effects on vaccine uptake were less clear due to public hesitancy. Drone delivery avoided ground-courier costs while maintaining cold-chain requirements and biohazard-safe packaging. The programme overcame initial public and expert skepticism to earn widespread commendation.

#### Geography and operational models

Drone programmes have been implemented in settings characterised by physical, seasonal, or infrastructural barriers to healthcare delivery. These include mountainous rural areas in Rwanda, island and lakeside communities in Malawi and Senegal [36], rainforest zones in the DRC [45], riverine terrain in Nigeria [42] and Malawi [39], semi-arid regions in Kenya [48], and remote rural or peri-urban areas in Ghana [29], Guinea [46], and Cameroon [44]. Across these contexts, the common feature is not simply rurality but the existence of transport bottlenecks that delay delivery of blood, medicines, vaccines, or diagnostic samples.

Most programmes use a hub-and-spoke architecture. Orders are placed from health facilities, commonly by phone or WhatsApp, processed at a central distribution hub, and dispatched either on demand or according to schedules. In many cases, health workers [23] collect the delivery from a designated landing or drop-off point rather than receiving it directly at the facility. This enables smaller, more responsive shipments than traditional bulk resupply models, but it also creates a distinct set of organisational tasks and dependencies [8,21,23,25].

The dominant implementing arrangement is a public-private partnership, usually involving international providers rather than locally owned operators. Zipline has become the most visible company in this field, operating in Ghana [8,21,23–35], Rwanda [40], Nigeria [41], Côte d’Ivoire [43], and Kenya [48]. Other models include VillageReach and Swoop Aero in Malawi [8,36] and the DRC [45], the DrOTS project in Madagascar [37], and smaller pilots using other platforms in Guinea [46] and Kenya [48].

Funding arrangements also vary by programme. Government-led or government-contracted arrangements dominate in Ghana’s [8,21–26,28–35] and Rwanda’s Zipline programmes [40,41], and the DRC’s Ministry of Health/VillageReach vaccine-transport partnership programme [45]. Multilateral and bilateral donor or philanthropic/NGO funding is more prominent in the Madagascar [37], Malawi [36], and Senegal pilots [27,38], and in Nigeria’s Gavi-supported vaccine programme [42]. Corporate and philanthropic contributions are also reported in Ghana [26,28] and Cameroon [26].

Fixed-wing autonomous drones are the most commonly reported type, particularly in Zipline programmes, and are well-suited to fast one-way delivery of small payloads over relatively long distances [25,28,35,41]. However, they generally lack vertical take-off and landing (VTOL) capability and therefore drop payloads by parachute rather than land. Hybrid or VTOL drones, used in Madagascar, Malawi, Senegal, and the DRC, can both deliver and retrieve items, making them more useful for sample referral and bidirectional logistics [36,37,39,45]. Other designs, including solar-powered and AI-enhanced systems, appear in isolated reports but have not yet been widely evaluated (31, 40).

#### Main applications of drones in healthcare

The most frequently reported use of drones in sub-Saharan Africa is the transport of blood and blood products. Studies from Ghana, Rwanda, Malawi, Kenya, and Guinea describe the delivery of red blood cells, plasma, platelets, cryoprecipitate, and rare blood groups in response to emergency or unpredictable demand [29,36,39–41,46,48]. This is an area where drones appear particularly well-suited, as transport delays can be clinically consequential, and some blood products are difficult to stock at peripheral facilities due to short shelf life and unpredictable demand.

A second major use is vaccines, including routine Expanded Programme on Immunisation vaccines, COVID-19 vaccines, and, more recently, malaria vaccines. These were reported in Ghana, the DRC, Nigeria, and elsewhere, often in connection with efforts to reduce stockouts, maintain cold-chain integrity, and improve access in remote communities [30,32,34,42,45]. In several settings, drone delivery was used for both routine replenishment and urgent top-ups when facilities were about to run out.

A third major use is for emergency medicines and urgent commodities, including oxytocin, antivenom, complicated malaria medicines, rabies prophylaxis, and treatments for eclampsia [21,22,24,29,36,48]. These products, like blood, are clinically valuable because they are time-sensitive and often unavailable at peripheral sites. In practice, however, some systems also delivered routine supplies and outpatient medicines, blurring the distinction between emergency and non-emergency uses and sometimes stretching systems beyond their original design logic [25,27,32].

The fourth major application is the transport of diagnostic samples, especially tuberculosis sputum samples, HIV early infant diagnosis samples, and COVID-19 swabs. These systems support laboratory referral where testing is centralised, and sample transport is otherwise slow or unreliable, as seen in Madagascar, Malawi, Senegal, Guinea, Ghana, and the DRC [23,36,37,45,46]. More experimental uses include vector surveillance through drone-mounted sensors and dissemination of public health messages during outbreak response, as reported in Madagascar, Senegal, and Uganda [38,47]. These emerging uses suggest that drones may have value beyond simple logistics, although the evidence remains sparse. Table 4 summarises the current drone applications by country in sub-Saharan Africa.

**Table 4.** Current drone applications by country in sub-Saharan Africa.

Country	Blood Products	Vaccines	Emergency Supplies	Routine Medicines	Diagnostic Samples	Other Healthcare Uses
Ghana	✔	✔	✔	✔	✔	✔
Madagascar				✔	✔	
Malawi	✔	✔	✔	✔	✔	✔
Rwanda	✔					
Cameroon	✔	✔		✔	✔	✔
Côte d’Ivoire	✔	✔		✔		
Democratic Republic of the Congo		✔			✔	✔
Guinea				✔	✔	
Kenya	✔	✔	✔	✔		
Nigeria	✔	✔		✔		
Senegal	✔	✔	✔	✔	✔	
Uganda						✔

Source: media and research reports (references in text).

#### Reported outcomes

The most consistent reported outcome is a reduction in delivery time. In Ghana, several studies report deliveries falling from hours to under 30 or 60 minutes [21,28,29]. In Rwanda, median drone delivery times for blood products were below 50 minutes and substantially shorter than estimated road delivery times [40]. Similar time gains were reported for diagnostic samples in Madagascar and Guinea and for vaccine transport in the DRC [37,45,46]. These time reductions are central to the rationale for drones, but they are only meaningful if they alter treatment delays, stock availability, or diagnostic turnaround in practice.

Several studies also reported improved access and coverage. Ghana’s distribution hubs were reported to serve between 2,000 and 3,200 facilities and to reach many hard-to-reach communities [22,30], while Nigeria’s Bayelsa State programme extended immunisation services to isolated riverine populations [42]. In the DRC, vaccine collection times fell dramatically, and availability rose from 65% to 98% in supported facilities [45]. In Ghana, drone-supported vaccine delivery was associated with improved immunisation coverage and fewer missed vaccination opportunities [30,32]. These findings suggest that drones can extend the effective reach of service, although the extent of benefit varies by geography, system design, and baseline transport alternatives.

Some studies reported improved health outcomes, but the evidence is more variable here. Ghanaian impact assessments described lower maternal mortality and higher antenatal attendance in drone-supported areas, while Rwandan studies linked improved access to blood products with lower postpartum haemorrhage and trauma mortality [29,32,41]. The DrOTS tuberculosis programme in Madagascar was associated with improved case finding and modelled reductions in TB-related deaths, and vaccine delivery models from Ghana suggested fewer childhood disease episodes and deaths averted [30,32,37]. These findings are encouraging, but they derive from heterogeneous study designs, including quasi-experiments, retrospective analyses, and modelling studies, so they should be interpreted as suggestive rather than definitive causal estimates.

Drone systems also appear to affect supply chain performance, especially by reducing stockouts and wastage. In Rwanda, blood product expirations fell sharply after the introduction of drones [40]. Ghanaian and Congolese studies reported reduced vaccine stockouts, fewer missed opportunities, and a shift away from overstocking towards more demand-responsive ordering [21,30,31,45]. However, several qualitative studies found that drones often revealed rather than solved deeper supply problems: where central distribution hubs lacked stock, faster delivery could not compensate for upstream shortages [8,25].

Acceptability was generally high, though not without qualification. Patients often expressed satisfaction with faster deliveries and improved service responsiveness, and many health workers valued reduced travel burdens and easier access to urgent supplies [21,29,42,45]. At the same time, initial scepticism, concerns about privacy, blood quality, data use, and technological ‘leapfrogging’ were reported in Rwanda, Ghana, Malawi, and Cameroon [26–28,35,39,40]. Acceptability was therefore generally positive but clearly shaped by broader perceptions of trust, governance, and fairness.

#### Implementation barriers and enabling conditions

Several technical and operational barriers recurred across studies. These included bad weather, strong headwinds, seasonal rainfall, and storms that could ground flights or require fallback to land or water transport [23,24,37,39]. Payload capacity limits, especially for fixed-wing drones, constrained what could be delivered in a single trip and sometimes required multiple flights [34]. Other difficulties included GPS interference, limited battery range, connectivity problems, and variable landing-site infrastructure [22,36]. The most robust systems mitigated some of these risks through redundancy, backup generators, spare power, multiple propellers, and improved battery systems, but such resilience requires investment and planning [25,41].

A second set of barriers was organisational. Drone systems often imposed extra work on frontline staff, who had to place orders, coordinate deliveries, and retrieve packages from landing points, sometimes at some distance from facilities [8,25]. In Ghana and Malawi, some programmes also appeared to contribute to an internal ‘brain drain,’ (see Box 1) as technically capable health workers moved into distribution-centre roles with higher pay or better working conditions [8]. In addition, systems designed for emergency deliveries were sometimes used heavily for routine supplies, undermining their responsiveness for genuine emergencies later in the month [25]. These findings suggest that drones change work processes and can generate new burdens even when they reduce transport delays.

The regulatory and policy environment was another major determinant of success. Rwanda and Ghana were repeatedly cited as relatively supportive environments, with clearer civil aviation pathways and political backing for health-related drone services [30,40,42]. Elsewhere, legal ambiguity, administrative delays, restrictive rules, or fragmented authority constrained implementation, as in Cameroon, Malawi, the DRC, and Côte d’Ivoire [24,39,43–45]. These studies suggest that successful scale-up depends on moving beyond ad hoc permissions towards clear, health-specific regulatory frameworks that address liability, privacy, airspace, and integration with health systems.

Finally, financing and sustainability remain unresolved challenges. Some modelling studies suggest drones can be cost-effective under particular assumptions, especially where transport delays are severe, and delivery speed is clinically important [32,37,46]. Yet qualitative studies reveal persistent concerns about value for money, donor dependence, and the long-term sustainability of systems with high fixed costs or narrow contracts [8,25,26,35]. In other words, drones may be cost-effective in some contexts and for some products, but the current evidence does not justify a blanket claim that they are always cost-saving or scalable without wider system investment.

### How can drones be used to solve other healthcare problems?

The literature is dominated by applications in infectious disease, maternal health, blood supply, and emergency logistics, reflecting the immediate priorities of many deprived health systems in SSA [29,32,34,40]. However, the operational logic of these systems — rapid, targeted, hub-based delivery to facilities that are otherwise difficult to reach — may also be relevant to other healthcare problems, including some aspects of non-communicable disease (NCD) care.

No included study directly evaluated a drone-supported NCD programme. Nevertheless, several findings suggest possible future relevance. Existing systems already deliver products and medicines that overlap with chronic or repeated care pathways, and they reduce time and travel burdens in ways that could matter for patients requiring regular medicine refills or repeat diagnostic testing [32,48]. HIV-related sample and medicine delivery offers a partial analogue for chronic disease logistics, although the organisational requirements of long-term NCD care differ from those of emergency or immunisation systems. The case for extending drone delivery to NCDs should therefore be treated as plausible but unproven, and as a priority for prospective implementation research rather than an established application.

The review also identified potential and emerging non-logistics uses. These include vector surveillance through drone-mounted sensors and the dissemination of public health messages in outbreak settings [38,47]. In Uganda, drones were used together with aerial mapping to support risk communication during the 2022–2023 Sudan virus disease outbreak in Kampala, helping contain the outbreak, though the response depended heavily on partner rather than domestic funding [47]. Although not yet demonstrated in SSA, drone-mounted imagery can resolve objects at a few centimetres’ resolution, finer than the satellite platforms that current vector-mapping studies in the region have used to characterise mosquito breeding habitats [38]. While still limited in scale, such applications indicate that the role of drones in health systems may extend beyond carrying commodities.

## Discussion

This rapid scoping review shows that drones are no longer a speculative technology in sub-Saharan African healthcare, but an established, if still unevenly distributed, operational tool. Across the 29 included studies, drones were most often used to transport blood products, vaccines, emergency medicines, and diagnostic samples in settings where geography, poor roads, or seasonal disruptions delayed conventional delivery. The strongest and most consistent finding is that drones can substantially shorten delivery times, especially for urgent products and facilities in remote or hard-to-reach areas. In several settings, this appears to have been accompanied by improved access to care, reductions in stockouts and wastage, and, in some studies, better health outcomes. At the same time, the evidence base remains heavily concentrated in a small number of countries, especially Ghana and Rwanda, and in a limited number of operational models, most notably, public–private partnerships involving large external providers such as Zipline [3,8,30,36,40].

A central implication of the review is that drones should not be understood primarily as a stand-alone technological innovation, but as part of a wider reorganisation of health logistics. Their apparent effectiveness depends not simply on aircraft performance, but on how they are embedded in supply chains, governance systems, and frontline routines. In Ghana and Rwanda, the reported gains in delivery timeliness and availability of blood and vaccines were made possible by centralised distribution hubs, digital ordering systems, contractual performance requirements, and relatively supportive regulatory environments [21,22,30,40,41]. Where these enabling conditions are absent, drones may still reduce transport time, but their health-system effect is likely to be weaker and less sustainable. This is particularly important because many of the reported benefits in the literature derive from the interactions between drone delivery and pre-existing weaknesses in road transport, blood banking, laboratory referral systems, and emergency logistics rather than from the technology alone [8,25].

The review also highlights an important paradox. Drones can solve some ‘last-mile’ delivery problems while simultaneously exposing deeper system bottlenecks upstream. Several studies from Ghana make clear that faster transport does not resolve shortages at distribution centres, weak procurement systems, poor electricity supply, or gaps in staffing at the facility level [8,25,29]. In other words, drones can improve connectivity without necessarily improving availability unless the broader logistics system can respond. This is perhaps the most important lesson for policy: drones are best seen as one component of a broader health system strengthening strategy, not as a substitute for it. Countries that deploy drones without strengthening central stock management, digital reporting, blood banking, cold-chain systems, or workforce capacity may create parallel logistics channels that are impressive but fragile [25,36,39,45].

A second major theme is that the benefits of drones are distributed unevenly. Several studies suggest that the largest time gains accrue to facilities furthest from conventional transport routes, but not all remote facilities benefit equally. In Rwanda, for example, hospitals closest to the drone hubs were better able to expand product range and respond to variable blood demand than those further away [41]. Similarly, in Guinea and Ghana, the greatest time savings were reported for the most remote facilities, while sites close to central laboratories or stores gained comparatively little [30,46]. This means that ‘equity’ claims require scrutiny. Drones may improve geographic equity in access to supplies, but only if hub placement, service contracts, payload design, and ordering rules are aligned with equity goals. Otherwise, they risk benefiting already better-connected facilities within a new aerial network. This is particularly relevant for policy-makers who may assume that any drone system automatically improves rural equity. The review suggests that design choices matter at least as much as deployment itself [30,41,42].

The evidence also varies by setting. Most included studies examined rural, remote, island, or riverine areas, where reported advantages were greatest [36,46,47], though rural location alone does not guarantee benefit. The value depends on demand, distance to hub, and alternative transport reliability. The smaller body of urban/peri-urban evidence points to different advantages: avoiding road congestion and accelerating laboratory sample referral, as illustrated by COVID-19 sample transport in Ghana [23] and HIV diagnostics in Conakry, Guinea [46]. Consequently, implementation decisions should be based on the specific transport bottleneck, not a simple rural–urban distinction.

It is also important to distinguish directly observed from projected outcomes. The most consistently observed effects were operational, including shorter delivery times, reduced travel, improved product availability, and stockout reduction [30,40,45]. Some studies also reported associations with immunisation coverage or clinical outcomes, though these designs rarely isolate drones from concurrent staffing or stock management changes. Other outcomes, including deaths averted, disease episodes prevented, and cost-effectiveness, were derived wholly or partly from decision models rather than direct observation [31,32,37], and depend on assumptions about coverage, cost, and the counterfactual transport system for their true policy-informing value.

The literature also suggests that drones have the greatest immediate value where demand is urgent, unpredictable, and difficult to meet through conventional stockholding. This explains why blood products, emergency obstetric medicines, anti-snake venom, and some vaccines have dominated current applications. These products combine high clinical value with transport sensitivity and often low tolerance for delay [29,36,40,41,44]. Diagnostic samples form a second major use case, especially where laboratory systems are centralised and turnaround times would otherwise be prolonged, as seen in Madagascar, Guinea, Malawi, Senegal, and Ghana [23,36,37,46]. These are settings in which speed, rather than sheer payload volume, is the critical performance criterion. This observation helps define where drone programmes may be most justifiable in the near term: they appear most valuable when transport delay is itself a major determinant of clinical risk or supply chain failure.

By contrast, the paper’s findings on future use for NCDs are necessarily more inferential. No included study directly evaluated a drone-based NCD programme. However, the review suggests that the operational logic of existing systems could plausibly be extended to some NCD-related functions, especially delivery of repeat medicines, transport of chronic-disease diagnostic samples, and support for peripheral clinics that currently lose time and staff to routine supply collection. The clearest analogue is the use of drones for antiretroviral medicines and other chronic or repeated treatments in settings where regular refill journeys are themselves a barrier to care [48]. That said, NCD management differs from blood or vaccine delivery in important ways: the volume of medicines is often larger, demand is more regular rather than emergency-driven, and adherence depends on continuity, counselling, and clinical monitoring rather than logistics alone. Any extension into NCD care should therefore be treated as a hypothesis for implementation research rather than as an established application [6,32,48].

The review further shows that the implementation of drone systems raises non-trivial workforce and governance questions. Drones may reduce some burdens, such as long-distance collection of blood or samples, but they also create new tasks for frontline staff, including ordering, coordination, repackaging, communication with distribution centres, and collection from drop-off points [8,23,25]. In some settings, private providers have also recruited health workers into distribution-centre roles, aggravating staffing shortages elsewhere in the system [8]. This underlines the need to consider drones not only as logistics infrastructure but also as labour-restructuring technologies. Their sustainability depends partly on whether new roles are clearly defined, staff are trained and supported, and the system does not simply shift workload from transport workers to already overstretched clinical staff [8,24,45].

Regulation is another decisive factor. The review confirms earlier work showing that [36] countries such as Rwanda and Ghana have benefited from more facilitative regulatory approaches, while others have faced delays, ambiguity, or fragmented oversight [36,39,40,42,48]. This matters for scale-up. Medical drone systems require more than aviation approval: they also need clarity about liability, sample handling, cold-chain standards, data protection, airspace access, and procurement rules. In conflict-affected or politically fragile settings, they may additionally face suspicion because drones are associated with surveillance or military uses [26,44]. The policy lesson is that governments and regulators need to move beyond ad hoc authorisations towards clearer health-specific frameworks for civil drone use, including provisions for public accountability, privacy protection, and safe integration with national logistics systems [36,39,45].

The findings on cost and cost-effectiveness should also be interpreted carefully. Some modelling studies suggest that drones can be cost-effective under specific assumptions, particularly where transport delays are severe and where reductions in stockouts, wastage, or treatment delays are large [32,37,46]. But the qualitative studies also reveal unease about value for money, especially when programmes rely on external subsidies, involve substantial fixed costs, or operate under emergency contracts that may not be efficient for routine supplies [8,25,26]. The review, therefore, does not support a simple conclusion that drones are ‘cost-saving’. Rather, they may be cost-effective in some contexts and for some products, but not in others. Better economic comparisons, including with strengthened road, boat, or motorcycle systems, are still needed. This is particularly important in low-resource settings, where choices about funding drones are also choices about what else cannot be funded.

### Implications for policy and implementation

Taken together, the findings suggest that drone programmes should be planned as part of a broader health-system strategy, not as isolated projects. First, deployment should target clearly defined problems, such as emergency blood delivery, vaccine resupply in remote areas, or rapid diagnostic referral, where drones offer a demonstrable advantage over ground transport. Second, investment should account for supporting conditions, including adequate central stock, digital ordering systems, trained staff, reliable power supply, and clear regulation. Third, equity should be built into service design through hub placement and prioritisation rules. Fourth, governments should avoid allowing donor-driven pilots to substitute for longer-term financing, procurement, and workforce planning [3,8,21,25].

Future expansion should be cautious but imaginative. The review supports continued use of drones where the case is strongest – blood, vaccines, selected emergency medicines, and diagnostic samples – while encouraging prospective trials or implementation studies in new areas such as NCD medicine delivery, chronic disease diagnostics, vector surveillance, and public health communication [38,47], grounded in local need, comparative evaluation, and realistic consideration of what drones can and cannot solve. They are unlikely to compensate for weak primary care, staff shortages, poor stock management, or broken referral systems on their own. Their value lies in complementing, not replacing, broader system investment.

### Limitations

The literature available for review in this rapidly developing field remains limited. Little attention has been paid to cost-effectiveness, scalability, generalizability to other countries, ethical issues, and the specific challenges arising in conflict zones. Standardised evaluation methods are needed to facilitate the comparability of studies. The existing literature pays little regard to community perspectives that must be taken into account in implementation. Although we searched for literature in English and French, we may have missed papers in Portuguese, Arabic, or African languages.

## Conclusion

Drones are no longer a speculative technology in sub-Saharan African healthcare. The evidence reviewed here shows that they are already being used to transport blood products, vaccines, emergency medicines, and diagnostic samples, with the clearest and most consistent benefit being substantial reductions in delivery time in remote and underserved settings [29,30,36,40].

At the same time, drones are not a stand-alone solution to weak health systems. Their value depends on the wider systems into which they are introduced, including central stock availability, digital ordering capacity, supportive regulation, workforce arrangements, reliable infrastructure, and sustainable financing. In several settings, drone programmes have exposed deeper bottlenecks in procurement, staffing, and supply chain governance rather than resolving them outright [8,25].

The strongest current case for drones lies in settings where delay is clinically consequential, especially for blood, vaccines, selected emergency medicines, and diagnostic referral. Their possible extension to non-communicable disease care is promising, but remains to be tested directly. Drones should therefore be seen as one component of broader health system strengthening, not a substitute for it [6,37,41,48].

**Table:** 

SSA	Sub-Saharan Africa
NCD	Non-communicable disease
EMS	Emergency medical services
AED	Automated external defibrillator
LMIC	Low- and middle-income countries
PCC	Population concept context
DRC	Democratic Republic of Congo
GPS	Global positioning system
UHC	Universal health coverage
VTOL	Vertical take-off and landing
LMD	Last mile distribution
DALY	Discounted disability-adjusted life year
DrOT	Drone-observed therapy system
DOTS	Directly observed therapy, short-course

## Abbreviations

## Supplementary Material

Supplementary_File_1_Search_Summary_Tables_clean.docx

Figure 1_legend.docx

Supplementary File 2_Filled PRISMA_ScR Checklist.pdf

## Data Availability

The data that support the findings of this study are openly available in Open Science Framework at https://osf.io/647wm/files/osfstorage.
