# Supplementary material for: Current and potential uses of drones in healthcare delivery in sub-Saharan Africa: a rapid scoping review
Source: Glob Health Action. 2026 Sep 1;19(1):2719410. doi: 10.1080/16549716.2026.2719410 (PMC13537061; doi:10.1080/16549716.2026.2719410)
Supplement: Supplementary file 2 [file ZGHA_A_2719410_SM5840.docx]

**Figure 1. PRISMA flow diagram of the study selection process.**

The diagram illustrates the systematic workflow of the scoping review process, detailing the number of records identified from each database; records screened, sought for retrieval, and assessed for eligibility; and records included in the analysis. The workflow for records identified, screened, and included from websites and through citation searching is also illustrated.
