# Supplementary material for: Current and potential uses of drones in healthcare delivery in sub-Saharan Africa: a rapid scoping review
Source: Glob Health Action. 2026 Sep 1;19(1):2719410. doi: 10.1080/16549716.2026.2719410 (PMC13537061; doi:10.1080/16549716.2026.2719410)
Supplement: Supplementary file 3 [file ZGHA_A_2719410_SM5841.docx]

# Supplementary File 1: Search Summary Tables

## Table 1: Search Summary Table for PubMed

| Search No. | Concept | Search String |
| --- | --- | --- |
| 1 | Drones | ("unmanned aerial devices"[MeSH] OR drone* OR "unmanned aerial vehicle*" OR "unmanned aircraft system*" OR "aerial delivery system*") |
| 2 | Medicine delivery | (medical OR medicine OR "Vaccines"[MeSH] OR vaccine* OR "Blood"[MeSH] OR blood OR "laboratory sample*") |
| 3 | Capacity | (capacity* OR efficien* OR performance* OR evaluat* OR delivery OR reliabilit*) |
| 4 | Sub-Saharan Africa / LMICs | ("Africa South of the Sahara"[MeSH] OR "Sub-Saharan Africa" OR "SSA" OR "Developing Countries"[MeSH] OR LMIC* OR "low- and middle-income countr*" OR "developing countr*") |

Search logic: #1 AND #2 AND #3 AND #4

Full PubMed search string:

("unmanned aerial devices"[MeSH] OR drone* OR "unmanned aerial vehicle*" OR "unmanned aircraft system*" OR "aerial delivery system*") AND (medical OR medicine OR "Vaccines"[MeSH] OR vaccine* OR "Blood"[MeSH] OR blood OR "laboratory sample*") AND (capacity* OR efficien* OR performance* OR evaluat* OR delivery OR reliabilit*) AND ("Africa South of the Sahara"[MeSH] OR "Sub-Saharan Africa" OR "SSA" OR "Developing Countries"[MeSH] OR LMIC* OR "low- and middle-income countr*" OR "developing countr*")

**Results = 37**

## Table 2: Search Summary Table for Scopus

| Search No. | Concept | Search String |
| --- | --- | --- |
| 1 | Concept 1: Drones | TITLE-ABS-KEY (drone* OR "unmanned aerial vehicle*" OR "unmanned aircraft system*" OR "aerial delivery system*") |
| 2 | Concept 2: Medical | TITLE-ABS-KEY (medical OR medicine OR vaccine OR blood OR "laboratory sample*") |
| 3 | Concept 3: Capacity | TITLE-ABS-KEY (efficien* OR performance* OR evaluat* OR delivery OR reliability) |
| 4 | Concept 4: LMICs / SSA | TITLE-ABS-KEY ("Sub-Saharan Africa" OR SSA OR LMIC* OR "developing countr*" OR "low- and middle-income country*") |

Full SCOPUS search string:

(TITLE-ABS-KEY (drone* OR "unmanned aerial vehicle*" OR "unmanned aircraft system*" OR "aerial delivery system*") AND TITLE-ABS-KEY (medical OR medicine OR vaccine OR blood OR "laboratory sample*") AND TITLE-ABS-KEY (efficien* OR performance* OR evaluat* OR delivery OR reliability) AND TITLE-ABS-KEY ("Sub-Saharan Africa" OR SSA OR LMIC* OR "developing countr*" OR "low- and middle-income country*"))

**Results = 41**

## Table 3: Search Summary Table for Web of Science

| Search No. | Concept | Search String |
| --- | --- | --- |
| 1 | Drones | TS=(drone* OR "unmanned aerial vehicle*" OR "unmanned aircraft system*" OR "aerial delivery system*") |
| 2 | Medical | TS=(medical OR medicine OR vaccine OR blood OR "laboratory sample*") |
| 3 | Capacity | TS=(efficien* OR performance* OR evaluat* OR delivery OR reliability) |
| 4 | LMICs / SSA | TS=("Sub-Saharan Africa" OR SSA OR LMIC* OR "developing countr*" OR "low- and middle-income country*") |

Full Web of Science search string:

TS=(drone* OR "unmanned aerial vehicle*" OR "unmanned aircraft system*" OR "aerial delivery system*") AND TS=(medical OR medicine OR vaccine OR blood OR "laboratory sample*") AND TS=(efficien* OR performance* OR evaluat* OR delivery OR reliability) AND TS=("Sub-Saharan Africa" OR SSA OR LMIC* OR "developing countr*" OR "low- and middle-income country*")

**Results = 12**

## Table 4: Search Summary Table for Google Scholar

| Search No | Concept | Search String |
| --- | --- | --- |
| 1 | Concept 1: Drones | (“drones” OR “unmanned aerial vehicles” OR “unmanned aircraft systems” OR “aerial delivery systems”) |
| 2 | Concept 2: Medical | (“medicine delivery” OR “medical supply delivery” OR “pharmaceutical transportation” OR “healthcare logistics” OR “medical courier services”) |
| 3 | Concept 3: Capacity | (“working capacity” OR “operational efficiency” OR “performance evaluation” OR “delivery effectiveness” OR “service reliability”) |
| 4 | Concept 4: LMICs / SSA | (“LMICs” OR “low- and middle-income countries” OR “developing countries” OR “resource-limited settings” OR “Global South” OR “Sub-Saharan Africa” OR “SSA” OR “Africa south of the Sahara”) |

Full Google Scholar search string:

(“drones” OR “unmanned aerial vehicles” OR “unmanned aircraft systems” OR “aerial delivery systems”) AND (“medicine delivery” OR “medical supply delivery” OR “pharmaceutical transportation” OR “healthcare logistics” OR “medical courier services”) AND (“working capacity” OR “operational efficiency” OR “performance evaluation” OR “delivery effectiveness” OR “service reliability”) AND (“LMICs” OR “low- and middle-income countries” OR “developing countries” OR “resource-limited settings” OR “Global South” OR “Sub-Saharan Africa” OR “SSA” OR “Africa south of the Sahara”)

**Results = 819**

## Table 5: Search Summary Table for Cairn.info

| Search No | Concept | Search String |
| --- | --- | --- |
| 1 | Drones | drone* OR UAV OR "véhicules aériens sans pilote" |
| 2 | Medical | santé OR medic* OR pharm* |
| 3 | Capacity | capacité OR efficacité OR performance OR évaluation OR fiabilité |
| 4 | LMICs / SSA | Afrique OR "pays en développement" OR "pays à revenu faible" OR "Sud global" |

**Full CAIRN.info search string:**

(drone* OR UAV OR "véhicules aériens sans pilote") AND (santé OR medic* OR pharm*) AND (capacité OR efficacité OR performance OR évaluation OR fiabilité) AND (Afrique OR "pays en développement" OR "pays à revenu faible" OR "Sud global")

**Results = 22**
